# The mediating role of state shame, guilt, and pride in the relationship between self-compassion and prolonged grief

**DOI:** 10.1192/j.eurpsy.2022.475

**Published:** 2022-09-01

**Authors:** H. Szőcs, L. Sandheden, Z. Horváth, G. Vizin

**Affiliations:** 1 Eötvös Lorand University Institute of Psychology, Department Of Clinical Psychology And Addiction, Budapest, Hungary; 2 Institute of Psychology, Doctoral School Of Psychology, Budapest, Hungary; 3 Eötvös Lorand University Institute of Psychology, Department Of Personality And Health Psychology, Budapest, Hungary; 4 Semmelweis University, Department Of Clinical Psychology, Budapest, Hungary

**Keywords:** prolonged grief, shame, self-compassion, pride

## Abstract

**Introduction:**

Most people experience grief-related symptoms after losing a loved one. Approximately 9.8% of bereaved individuals’ symptoms persist over the first year post-loss, emphasizing the importance of research in prolonged grief. The role of self-conscious emotions in prolonged grief, such as self-compassion, state shame, guilt and pride has been proposed in previous studies.

**Objectives:**

Our aim was to examine the mediating role of state shame, guilt and pride in the relationship between self-compassion and prolonged grief.

**Methods:**

This cross-sectional study collected data via online questionnaires based on self-reports (N=177, mean age: 42.26 years [SD=12.32], 97.2% women). Demographic and loss-related variables were measured, and further instruments assessed levels of self-compassion, state shame, guilt, and pride, and prolonged grief. Correlation and mediation analyses were used.

**Results:**

Two significant indirect effects were shown in the mediation model. First, lower levels of self-compassion predicted higher levels of state shame, which in turn predicted elevated levels of prolonged grief. Second, higher levels of self-compassion predicted higher levels of pride, which subsequently contributed to lower levels of prolonged grief. Guilt did not have a significant mediating role.

**Conclusions:**

The results highlight the possible role of elevated levels of state shame and lower levels of self-compassion and state pride in the development of prolonged grief. It is important for researchers and clinicians to be attentive to the role of self-compassion, state shame and pride in grieving.

**Disclosure:**

No significant relationships.

